# Successful dupilumab treatment in a patient with severe dermatitis following allogenic hematopoietic stem cell transplantation

**DOI:** 10.1186/s13223-025-00966-3

**Published:** 2025-04-29

**Authors:** Young-Hee Nam, Hyun Jung Jin

**Affiliations:** 1 Department of Internal Medicine, College of Medicine, Dong-A University, Busan, Korea; 2 Department of Internal Medicine, Yeungnam University College of Medicine, Daegu, Korea

**Keywords:** Atopic dermatitis, Dupilumab, Hematopoietic stem cell transplantation

## Abstract

**Background:**

Allogenic hematopoietic stem cell transplantation (HSCT) is the optimal treatment of hematologic diseases and various malignancies. Development of allergic disease in a transplant patient has been reported.

**Case presentations:**

A 49-year-old male with no history of atopy underwent two allogenic HSCTs for aplastic anemia from his brother with severe atopic dermatitis 11 years ago. The patient developed eczema on whole body and an elevated peripheral blood eosinophil count of 5775 cells/µL at 3 months after the second HSCT. Despite prolonged treatment with systemic corticosteroids and immunomodulators the skin rash and elevated blood eosinophil count persisted. However, after 4 months of dupilumab therapy, the patient showed near-complete clearance of symptoms. The sustained clinical improvement was observed during 36 months treatment without adverse drug reactions.

**Conclusions:**

Although rare, atopic dermatitis can occur after HSCT, and dupilumab may be safe and effective for refractory conditions.

## Background

Allogenic hematopoietic stem cell transplantation (HSCT) is an established treatment for hematologic diseases, including aplastic anemia, genetic disorders and various malignancies. Transfer of allergic diseases such as rhinitis, conjunctivitis, and asthma from an atopic donor to a HSCT recipient has been reported [1–4]. In contrast, other allergies have been resolved after receiving HSCT [5, 6]. The recipients either acquired or developed an exacerbation of allergic diseases such as conjuntivitis, asthma, food allergy and urticaria. We present the case of a patient who developed atopic dermatitis (AD) after HSCT and was successfully treated with dupilumab despite conventional immunosuppressive therapy.

## Case presentation

A 49-year-old male presented with eczematous and crusted erythematous rashes on whole body. The patient had undergone an allogeneic HSCT for aplastic anemia from his younger brother 12 years ago. The patient received standard post-transplantation immunosuppression therapy with cyclosporine for 14 months, continuous methotrexate, and systemic steroids for 16 months. However, aplastic anemia relapsed, and he received a second allogenic HSCT from the same donor at 20 months after the first HSCT. He had no history of acute or chronic graft-versus-host disease.

At 3 months after the second HSCT, peripheral blood eosinophil count of the patient increased up to 5775 cells/µL, and he experienced erythematous, scaly, pruritic rashes on his legs. Serum total IgE levels were normal (95.4 kU/L). Topical corticosteroids and topical calcineurin inhibitors were ineffective. Additionally, systemic therapies including corticosteroids and cyclosporine were continued for 21 and 16 months, respectively. However, the widespread eczematous lesions extended to the arms, trunk, neck, and face, and progressively worsened. A persistent increase in blood eosinophil levels (10–20%) was observed. Systemic corticosteroids were frequently administered to control cutaneous symptoms for 11 years, and the treatment intervals had been gradually shortened.

**Fig. 1:**
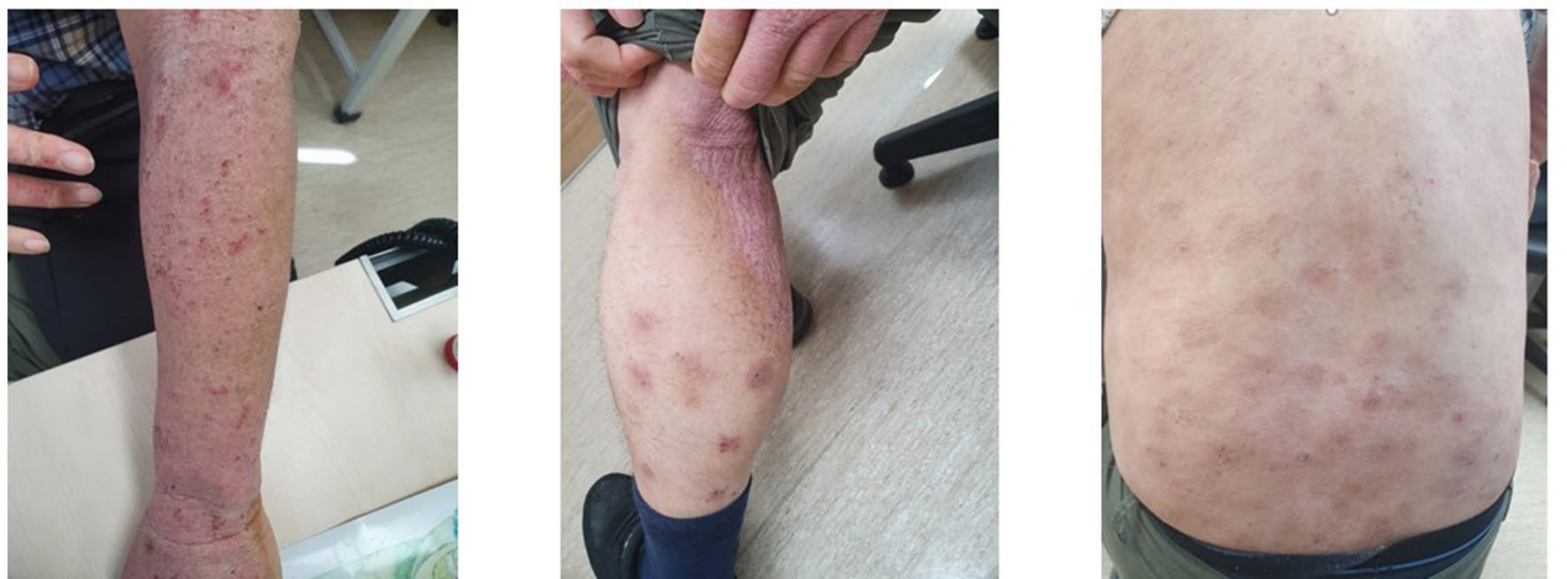
The patient showed the diffused erythematous scaly eczematous rash on the entire body before dupilumab treatment

**Fig. 2:**
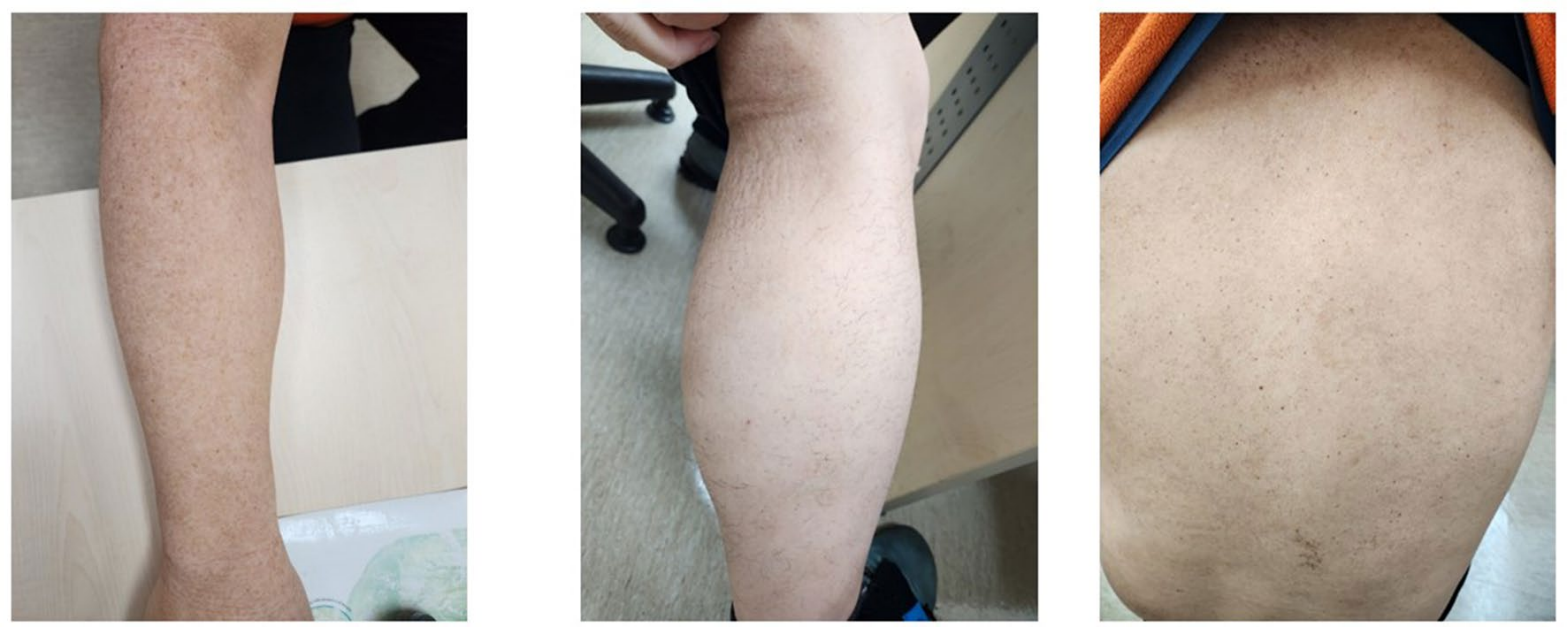
Skin rash was nearly resolved after four months of dupilumab therapy, with persistent improvement of documented at one year of treatment

The patient had no history of allergic disease before HSCT; however, the donor sibling had developed AD in adulthood. The recipient presented with severe AD involving nearly the entire body, with an eczema area and severity index (EASI) score of 27 (Fig. 1) on his first visit to our clinic. Initial treatment with methotrexate for 4 months for severe AD resulted in no clinical response (EASI score, 24.9). Total IgE (> 5000 kU/L) and peripheral blood eosinophil count (3162 cells/µL) were elevated. The patient was started on dupilumab therapy at 300 mg every two weeks after a loading dose of 600 mg. His eczema and pruritus improved after 1 month of dupilumab treatment (EASI score, 9.2) and achieved marked and gradual improvement at 4 months of treatment (EASI score, 1.2). After 6 months on dupilumab, total IgE levels (2386 kU/L) and eosinophil counts (806 cells/µL) decreased and near-complete resolution was achieved (EASI score, 0.8). The patient is still on continuous duilumab therapy for 36 months, and maintains sustained improvement in skin lesions (Fig. 2) without clinical deterioration and adverse drug reactions. He uses only emollients for dry skin management.

## Discussion and conclusion

AD is a chronic, heterogeneous, and inflammatory skin disorder characterized by diverse clinical phenotypes and courses. The pathophysiological mechanisms of AD involve complex interactions between genetics, immune system dysregulation, and environmental factors. AD is predominantly Th2 driven, and other cellular subsets such as Th1, regulatory T cell, Th17, and Th22 cells contribute to immune activation [7]. Development of allergic diseases including AD in a patient received HSCT also has been reported [1–3, 8–10]. Th2-biased immune reconstitution after HSCT has been suggested. It could be the acquisition of T cells in recipients are biased toward Th2-screwed immune response due to genetic and/or environmental factors in the donors. After HSCT, mature memory cells are replaced by cells derived from donor hematopoietic progenitor cells [11]. Marrow-derived immune cells from allergic donors may transfer allergic diseases [1, 12]. Th2 cytokine like interleukin (IL)-4 and IL-10 may be up-regulated in HSCT recipients [13]. In addition, systemic tacrolimus for post-transplantation immunosuppression was associated with Th1/Th2 imbalance with predominance of Th2 response and was implicated as a potential trigger of allergic disease [14–15]. However, sustained Th2-biased response as a consequence of HSCT is unlikely because allergic disease is not commonly reported after HSCT. The patient in this case did not receive tacrolimus for GVHD prophylaxis, and AD might occur sporadically after HSCT.

Dupilumab is a monoclonal antibody that blocks IL-4 and IL-13, the key drivers of Th2-mediated inflammation. It was the first biologic approved for moderate-to-severe AD. Dupilumab has demonstrated efficacy and safety in clinical trials and real-life clinical settings. Several cases of AD or AD-like graft-versus-host disease (GVHD) that develop after HSCT have been successfully treated with dupilumab [16–18]. In previous reports [16–18], eczematous dermatitis developed usually between 1 and 5 months after HSCT. Dupilumab therapy showed a rapid clinical response after 1 month, with nearly complete clearance observed after 4 months, and the discontinuation of other immunosuppressive therapies. The patient in this case experienced AD 3 months after HSCT and was refractory to systemic immunosuppressants, such as corticosteroids, cyclosporine, and methotrexate. However, the patients showed complete resolution of the cutaneous manifestations, and systemic immunosuppressants were discontinued after dupilumab therapy. To our knowledge, this patient had the longest duration of follow-up reported for dupilumab therapy (36 months), without disease flares or adverse reactions. AD-like GVHD, a subtype of GVHD, occurs after HSCT and is similar to de novo AD in terms of clinical and histological features, including a predominant Th2 immune response [19, 20]. Therefore, distinguishing AD-like GVHD from other cutaneous disorders, including AD, eczematous GVHD subtype, or eczematous dermatitis, can be challenging. Patients with AD-like GVHD have clinical features usually characterized by no atopic history, no donor-related atopy, and a good response to conventional treatments, such as systemic corticosteroids and immunosuppressants [20, 21]. However, the patient was refractory to conventional treatments, and the donor had AD prior to transplantation. More than two dermatologists observed the patient for 11 years after the onset of eczema. Finally, the patient was diagnosed as AD based on the clinical evaluations, although skin biopsy was not performed.

AD can develop after HSCT, and dupilumab shows rapid, long-term efficacy and safety in refractory cases.

## Data Availability

No datasets were generated or analysed during the current study.

## Abbreviations

HSCT: Hematopoietic stem cell transplantation
IgE: Immunoglobulin E
AD: Atopic dermatitis
EASI: Eczema area and severity index
GVHD: Graft-versus-host disease
